# Prenatal drinking-water exposure to tetrachloroethylene and ischemic placental disease: a retrospective cohort study

**DOI:** 10.1186/1476-069X-13-72

**Published:** 2014-09-30

**Authors:** Jenny L Carwile, Shruthi Mahalingaiah, Michael R Winter, Ann Aschengrau

**Affiliations:** Department of Epidemiology, Boston University School of Public Health, Boston, MA USA; Department of Obstetrics and Gynecology, Boston University School of Medicine, Boston, MA USA; Data Coordinating Center, Boston University School of Public Health, Boston, MA USA

**Keywords:** Drinking-water, Ischemic placental disease, Placental abruption, Preeclampsia, Small for gestational age, Stillbirth, Tetrachloroethylene

## Abstract

**Background:**

Prenatal drinking water exposure to tetrachloroethylene (PCE) has been previously related to intrauterine growth restriction and stillbirth. Pathophysiologic and epidemiologic evidence linking these outcomes to certain other pregnancy complications, including placental abruption, preeclampsia, and small-for-gestational-age (SGA) (i.e., ischemic placental diseases), suggests that PCE exposure may also be associated with these events. We examined whether prenatal exposure to PCE-contaminated drinking water was associated with overall or individual ischemic placental diseases.

**Methods:**

Using a retrospective cohort design, we compared 1,091 PCE-exposed and 1,019 unexposed pregnancies from 1,766 Cape Cod, Massachusetts women. Exposure between 1969 and 1990 was estimated using water distribution system modeling software. Data on birth weight and gestational age were obtained from birth certificates; mothers self-reported pregnancy complications.

**Results:**

Of 2,110 eligible pregnancies, 9% (N = 196) were complicated by ≥1 ischemic placental disease. PCE exposure was not associated with overall ischemic placental disease (for PCE ≥ sample median vs. no exposure, risk ratio (RR): 0.90; 95% confidence interval (CI): 0.65, 1.24), preeclampsia (RR: 0.36; 95% CI: 0.12-1.07), or SGA (RR: 0.98; 95% CI: 0.66-1.45). However, pregnancies with PCE exposure ≥ the sample median had 2.38-times the risk of stillbirth ≥27 weeks gestation (95% CI: 1.01, 5.59), and 1.35-times of the risk of placental abruption (95% CI: 0.68, 2.67) relative to unexposed pregnancies.

**Conclusions:**

Prenatal PCE exposure was not associated with overall ischemic placental disease, but may increase risk of stillbirth.

**Electronic supplementary material:**

The online version of this article (doi:10.1186/1476-069X-13-72) contains supplementary material, which is available to authorized users.

## Background

In New England between the late 1960’s and early 1980’s, drinking water exposure to PCE (perchloroethylene or tetrachloroethylene) was relatively common as the result of the installation of vinyl-lined asbestos cement (VL/AC) pipes within public water systems. The vinyl lining, a slurry of PCE and vinyl toluene resin (Piccotex; Johns-Manville Corporation, Denver, CO), was introduced as a solution to taste and odor problems found in existing pipes. PCE was expected to evaporate by the time of pipe installation; however, testing in 1980 revealed that large quantities of PCE remained in public drinking water supplied by VL/AC pipes. Although this specific source of contamination has since been remediated by bleeding and flushing VL/AC pipes, adverse health consequences have also been attributed to public water sources contaminated by PCE from landfills and underground storage tanks [1], industrial waste disposal sites [2, 3], and dry cleaning facilities [4]. Thus, community-based PCE exposure remains an important topic of continued investigation.

Prenatal drinking water exposure to PCE has been previously linked to stillbirth [2] and intrauterine growth restriction [2, 4], and investigation of related disorders, including placental abruption and preeclampsia, appears warranted. These four outcomes, sometimes termed ischemic placental diseases or placental dysfunction disorders [5–7], represent a heterogeneous group of disorders affecting placental function and fetal growth which are thought to have a common etiologic pathway at the level of placental vascular development [8]. Ischemic placental diseases may also share common risk factors [9–13], making knowledge of risk factors for one ischemic placental disease potentially informative for identifying novel risk factors for related disorders.

Based on this, we examined the association between prenatal PCE exposure and ischemic placental disease in a population-based retrospective cohort of 1,766 women.

## Methods

### Study population and follow-up

The Cape Cod Family Health Study is a population-based retrospective cohort study initiated to examine the influence of prenatal exposure to PCE-contaminated drinking water on multiple outcomes during pregnancy and childhood [14–16]. The study was approved by the Institutional Review Boards of the Massachusetts Department of Public Health, Boston University Medical Center, and the 24A/B/11B Review Committee at the Massachusetts Department of Public Health.

Women were considered eligible for the parent cohort if they gave birth to at least one child (identified here as “index” pregnancies or children) between 1969 and 1983 and were living in one of eight Cape Cod towns with some documented VL/AC water distribution pipes at the time of the child’s birth. Eligibility was determined by cross-matching the maternal address on the birth certificate with water distribution system data obtained from local water department records. For the initial exposure assessment, we classified women as “exposed” or “unexposed” at the time of the index child’s birth based on visual inspection of pipe distribution maps in the vicinity of the maternal address on the birth certificate. We considered 1,492 women to be exposed at the time of the index child’s birth because their residence was either a) adjacent to a VL/AC pipe or b) adjacent to a non-VL/AC pipe connected to a VL/AC pipe and this was the only possible water flow to the residence. From the remaining eligible women, we selected a comparison group of 1,704 unexposed women frequency matched to exposed women based on the birth month and year of the index child. Initial exposure status was considered preliminary, and final exposure classification incorporated more detailed exposure assessment, as described below, and was performed with respect to each pregnancy.

Between 2002 and 2003, we attempted to obtain the current address and telephone number for eligible mothers, or fathers, if the mother was deceased. Introductory letters and self-administered questionnaires were sent to all traced participants. If a participant failed to respond to our initial contact, we made two additional attempts to reach them by mail, and if that was also unsuccessful, we attempted to contact them by phone.

Similar response rates were observed for women initially classified as exposed and unexposed (Table 1). Participants and non-participants were similar with respect to race and birth year of the index child; however, non-participating women were younger, less educated, and had more prior births than participating women. The gestational duration of index pregnancies was similar, but non-participating women gave birth to somewhat lighter infants than participating women. Notably, these differences were observed for both exposed and unexposed participants [14, 15].

**Table 1:** **Selection and enrollment status of Cape Cod, Massachusetts women contributing at least one index pregnancy, by initial exposure status [N (%)]**

		Initial exposure status ^a^	
	Exposed ^b^	Unexposed ^c^	Total
Selected	1,492	1,704	3,196
Excluded during enrollment			
Never located	132 (8.8)	136 (8.0)	268 (8.4)
No response	245 (16.4)	336 (19.7)	581 (18.2)
Ineligible	7 (0.5)	8 (0.5)	15 (0.5)
Refusal	149 (10.0)	137 (8.0)	286 (8.9)
Returned questionnaire			
Percent of located	959 (70.5)	1,087 (69.3)	2,046 (69.9)
Percent of selected	959 (64.3)	1,087 (63.8)	2,046 (64.0)
Excluded during exposure assessment and analysis			
Incalculable exposure—insufficient data for geocoding residential address	60 (6.3)	75 (6.9)	135 (6.6)
Incalculable exposure-missing data on LMP	20 (2.1)	21 (1.9)	41 (2.0)
Multiple birth	11 (1.2)	21 (1.9)	32 (1.6)
Birth anomaly	22 (2.3)	32 (2.9)	54 (2.6)
Missing data on key covariates	9 (0.9)	9 (0.8)^d^	18 (0.9)
Final sample	834	932	1,766

*Abbreviation:* *LMP* last menstrual period.

^a^The exposure status of each pregnancy was assessed later (see Methods).

^b^Contributed ≥1 exposed index births.

^c^Contributed no exposed index births.

^d^One woman contributed a stillbirth only to the final analyses.

### PCE exposure assessment

We assigned each woman an initial exposure classification based on a visual inspection of the proximity of her residence to VL/AC pipes, as described above. Later, we refined our approach using the Webler and Brown leaching and transport model [17] to estimate the mass of PCE that was delivered to each residence of interest, from which we calculated a woman’s PCE exposure before and during each pregnancy. This model uses data on specific pipe characteristics to estimate the quantity of PCE entering the drinking water. Specific variables include the initial PCE loading in the pipe liner, estimated using pipe dimensions; the age of the pipe; and the leaching rate of PCE from the liner into the drinking water [18]. The model also requires data on water flow and direction estimated using the configuration of the distribution system and the number of water users. For this, we incorporated the Weber and Brown algorithm into EPANET water distribution system modeling software (http://www.epa.gov/nrml/wswrd/dw/epanet.html), which was developed by the U.S. Environmental Protection Agency and has been used to estimate exposure to drinking water contaminants in multiple epidemiologic studies (e.g., [19]).

As part of the self-administered questionnaire, women reported the calendar year of occupancy, exact street address and nearest cross street, and drinking water source for each of their family’s Cape Cod residences between 1969 and 1990. Each reported residence was incorporated into a geographic information system (GIS) using ArcGIS 8.1 (ESRI, Redlands, CA). Geocoding was conducted without knowledge of exposure status or pregnancy history. The majority of the 5,324 total reported addresses were successfully geocoded to a parcel of land (87.6%), or, in instances in which the street number was missing, the nearest reported cross street or the middle of the street (9.6%). Pregnancies associated with the remaining 2.7% of addresses that were unable to be geocoded were excluded from analyses. We assumed that all land parcels represented water users, all water users in the network drew the same quantity of water, and water sources did not change over the study period. These assumptions are supported by observations that the study area was mainly comprised of residences, and the distribution system changed little between the late 1960s and late 1980s, when some water sources were added to accommodate population growth. In addition to the geocoded residences, the GIS schematic depicted nodes, or points of water consumption, and pipe characteristics, which we inputted using data on the location, installation date, and diameter of VL/AC pipes obtained from local water companies and the Massachusetts Department of Environmental Protection (DEP; Boston, Massachusetts). The GIS represented the pipe configuration present around 1980. Lastly, each residence was assigned to the closest node on the water distribution system. We considered residences served by a private well or located in Cape Cod towns with no VL/AC pipes to have no PCE exposure during the study period.

For each pregnancy of interest, we estimated two measures of PCE exposure: cumulative exposure before pregnancy and average monthly exposure around the time of conception. We estimated cumulative exposure before pregnancy by summing the annual mass of PCE (g) for each residence between the move-in year or VL/AC pipe installation year, whichever came later, and the month and year of the last menstrual period (LMP). Because we did not have data on the month of move-in or VL/AC pipe installation, we estimated the annual PCE exposure or, for exposure occurring only a portion of the year, took a simple percentage of the annual exposure. We estimated the average monthly exposure during the LMP year by dividing annual exposure during the LMP year by 12. We estimated the LMP using data from birth certificates or questionnaires. The LMP and first trimester occurred in the same year for the majority of pregnancies (85%). In instances in which this was not the case, misclassification was likely modest as annual exposure levels between the LMP and first-trimester years were highly correlated (Pearson correlation coefficient = 0.96; *P* < 0.0001).

Both exposure metrics represent the estimated mass of PCE delivered to a residence during a specific time period, only a small portion of which was actually consumed by study participants. After taking into account dilution in the estimated 90,000 gallons of water used annually by an average Massachusetts household [20], we converted the estimated PCE mass delivered to a residence to an average annual point concentration. These values were consistent with actual water-sampling data from that time period [18].

### Assessment of ischemic placental disease

Between 2002–2003, we used birth certificates and questionnaires completed by the women to obtain data on pregnancies occurring between 1969 and 1983 in any of the eight Cape Cod towns of interest (i.e., “index” pregnancies). Data on all other pregnancies contributed by these women (i.e., “non-index” pregnancies) were obtained from the questionnaire only. Thus, women could contribute only index pregnancies or both index and non-index pregnancies. Women reported whether each pregnancy ended in a live birth, stillbirth, miscarriage, abortion (induced or therapeutic), or ectopic pregnancy, as well as the date that the pregnancy ended, and the approximate length of the pregnancy. Women also reported whether pregnancies were complicated by placental abruption or separation, preeclampsia, or vaginal bleeding. The gestational age at which any complications occurred was not obtained. Data on birth weight was obtained from birth certificates to limit misclassification. We investigated the following individual ischemic placental diseases: placental abruption, preeclampsia, small for gestational age (SGA) infant (birth weight <10th percentile of a gestational age- and sex-specific birth weight distribution for singleton births) [21], and stillbirth. We also investigated vaginal bleeding at any gestational age, which has a more heterogeneous etiology, but may be linked to placental dysfunction. Finally, we constructed a composite outcome of overall ischemic placental disease, which included pregnancies with ≥1 of the following: placental abruption, preeclampsia, an SGA infant, or stillbirth.

Of 6,519 total pregnancies, we excluded pregnancies with an unknown outcome (N = 14), ectopic pregnancies (N = 49), and abortions (N = 367). We additionally excluded pregnancies with incalculable exposure status due to missing data on LMP (N = 178) or insufficient residential data for accurate geocoding and exposure assessment (N = 344), as well as losses <27 weeks gestation (N = 623) and multiple births (N = 72). After further restriction to pregnancies without missing data on key covariates, 4,760 pregnancies were eligible for individual analyses of placental abruption, and 4,836 pregnancies (4,809 live births and 27 stillbirths) were eligible for analyses of preeclampsia and stillbirth. An eligibility flowchart shows this in more detail (see Additional file 1). We obtained data on birth weight and gestational duration from birth certificates, which were only available for index children; thus, for analyses of the composite outcome, we further restricted the study population to 2,110 pregnancies (2,084 index live births and 26 stillbirths) with no reported birth anomalies (excluding 2,542 non-index births, 171 babies with birth anomalies, and 13 observations missing data on key covariates). Of these, only live births were considered eligible for individual analyses of SGA (N = 2,002 after further exclusion of 82 with missing data on covariates).

### Assessment of covariate data

Data on the parents’ ages and educational levels were obtained from the birth certificate for index children, and from the self-administered questionnaire for non-index children. Other data on parental demographics and reproductive characteristics were also obtained from the questionnaire.

### Statistical analysis

We calculated risk ratios (RR) and 95% confidence intervals (CI) for the association between prenatal PCE exposure and ischemic placental disease using log-binomial regression. To account for the non-independence of pregnancies contributed by the same woman, we used generalized estimating equations (GEE) [22, 23], for which we assumed constant correlation between pregnancies contributed by the same mother. For our primary approach, we compared pregnancies with PCE exposure ≥ the sample median or < the sample median to those with no exposure, which was considered the reference group. We also compared pregnancies with any PCE exposure to those with no exposure. For analyses of the composite outcome, we examined PCE exposure in two additional ways: 1) to investigate the dose–response of the association, we categorized PCE exposure into quartiles and compared pregnancies in each quartile of exposure to the reference group of no exposure, and 2) we classified exposed pregnancies with respect to levels corresponding to the 1980 action level of 40 ppb (1.136 g) which prompted environmental remediation [18] and compared each of these groups (≥40 ppb and <40 ppb) to unexposed pregnancies. Small sample size precluded us from utilizing these additional exposure metrics for analyses of individual ischemic placental diseases.

We conducted multivariable analyses adjusting for previously identified risk factors for individual ischemic placental diseases. Women with previous ischemic placental disease are at an increased risk of developing ischemic placental disease during subsequent pregnancies [5, 24–27]; thus, whenever possible, we adjusted for the potentially important confounder of prior ischemic placental disease. Models for placental abruption were adjusted for hypertension before or during pregnancy (yes, no) and first-trimester smoking (none, <11 cigarettes/day, ≥11 cigarettes/day) [28]; models for preeclampsia were adjusted for first-trimester smoking and combined parity and prior ischemic placental disease (nulliparous, ≥1 prior pregnancy with no prior ischemic placental disease, ≥1 prior pregnancy with prior ischemic placental disease) [29]; models for SGA were adjusted for maternal age (<25 y, 25–29.9 y, 30–34.9 y, ≥35 y), year that the pregnancy ended (<1974, 1975–1980, and >1980), gestational weight gain <20 lb (yes, no), hypertension before or during pregnancy, combined parity and prior ischemic placental disease, and first-trimester smoking [30–32]; models for stillbirth were adjusted for first-trimester smoking [33]; and models for vaginal bleeding were adjusted for maternal age and combined parity and prior ischemic placental disease [34, 35]. Based on this, we adjusted models for overall ischemic placental disease for the year that the pregnancy ended, maternal age, and combined parity and prior ischemic placental disease. When we individually added additional covariates, including first-trimester smoking, inadequate weight gain, and hypertension before or during pregnancy, to the model, none were found to have a meaningful impact on observed effect estimates.

As a sensitivity analysis, we examined associations of prenatal PCE with placental abruption, preeclampsia, and stillbirth in the restricted sample used for the composite outcome (e.g., only index live births and losses). We investigated whether associations varied by maternal age, prior ischemic placental disease, year of pregnancy, or first trimester cigarette smoking or alcohol use.

## Results

A total of 2,110 pregnancies, including 2,084 live index births for whom we obtained birth certificate data and 26 stillbirths (≥27 weeks gestation), were considered eligible for analyses of the composite outcome. This included pregnancies from 1,766 women, 300 (17%) of whom contributed 2 pregnancies, and 44 (2%) of whom contributed ≥3 pregnancies. We classified 196 (9%) of these pregnancies as having at least one ischemic placental disease, with SGA the most commonly identified disorder (N = 133; 6% of live births). Placental abruption (N = 20), preeclampsia (N = 25), and stillbirth at ≥27 weeks gestation (N = 26) were each identified in approximately 1% of eligible pregnancies. The majority of affected pregnancies were characterized by a single disorder (96%); 8 pregnancies were complicated by ≥1 disorder. Approximately 6% of live births complicated by ischemic placental disease resulted in a preterm birth (N = 10).

Many parental characteristics, including maternal age, race, and education, were similarly distributed across PCE-exposed and unexposed pregnancies (Table 2). However, certain groups, including parous women and women whose spouses were employed in a white-collar occupation, were more likely to have an exposed pregnancy.

**Table 2:** **Characteristics of 2,110 index pregnancies, by PCE exposure during the LMP year and occurrence of ischemic placental disease** ^**a**^

Characteristic		Ischemic placental disease		No ischemic placental disease
	% Exposed (N = 92)	% Unexposed (N = 104)	% Exposed (N = 999)	% Unexposed (N = 915)
Year of pregnancy^b^				
Before 1974	15.2	21.2	15.7	18.3
1975-1980	51.1	44.2	49.8	49.4
After 1980	33.7	34.6	34.5	32.4
Maternal age (y)^c^, mean (SD)	26.2 (4.3)	26.6 (4.4)	27.5 (4.5)	27.0 (4.6)
Paternal age (y), mean (SD)	29.5 (5.8)	29.5 (5.5)	30.7 (5.6)	29.9 (5.6)
White	91.3	95.2	95.7	97.4
Maternal education^b^				
Less than high school	3.3	1.9	1.1	1.1
High school graduate	22.8	25.0	19.2	18.4
Some college	32.6	35.6	33.9	34.8
Four-year college graduate or more	41.3	37.5	45.8	45.8
Paternal occupation^b^				
White collar	40.2	39.4	53.7	46.0
Blue collar	34.8	42.3	31.6	32.0
Other	21.7	15.4	13.2	20.4
Number of prior livebirths and prior ischemic placental disease^b^				
Nulliparous	43.5	58.7	35.5	41.0
Previous pregnancy, no prior ischemic placental disease	45.7	35.6	60.1	56.7
Previous pregnancy, prior ischemic placental disease	10.9	5.8	4.4	2.3
Diabetes before or during pregnancy	1.1	4.8	2.3	2.2
Hypertension before or during pregnancy	10.9	18.3	5.2	5.8
Gestational weight gain <20 lb	30.4	21.2	13.8	12.4
Cigarette smoking during first trimester	41.3	36.5	24.8	27.2
Alcohol consumption during first trimester	32.6	40.4	37.2	37.6
Occupational exposure to solvents^b^				
No	76.1	86.5	86.3	87.2
Yes, before or during pregnancy	17.4	11.5	10.9	9.8
Yes, unknown when	2.2	1.0	1.7	1.3
Use of solvent-based spot removers	63.0	57.7	67.3	64.0
Use of professional dry cleaning	82.6	86.5	87.7	85.8
Use of self-service dry cleaning	21.7	13.5	13.8	16.7

*Abbreviations:* *LMP* last menstrual period, *PCE* tetrachloroethylene, *SD* standard deviation.

^a^Including placental abruption, preeclampsia, small for gestational age, and stillbirth at ≥27 weeks gestation.

^b^Percentages do not sum to 100 due to rounding or missing values.

^c^Missing: paternal age (N = 18), paternal occupation (N = 35), diabetes (N = 20), hypertension (N = 23), gestational weight gain <20 lb (N = 62), alcohol consumption (N = 4), occupational exposure to solvents (N = 31), use of spot removers (N = 40), use professional dry cleaning (N = 79), use of self-service dry cleaning (N = 56).

Average monthly PCE exposure during the LMP year ranged from 0 to 89.2 g, with a median of 0.9 g among exposed pregnancies (Table 3). Cumulative PCE exposure before the LMP month and year ranged from 0 to 3,600 g, with a median of 28.9 g among exposed pregnancies. The two measures were highly correlated, with a Spearman correlation coefficient of 0.88 (*P* < 0.001).

**Table 3:** **Distribution of cumulative PCE exposure before LMP and average monthly PCE exposure during LMP year among exposed index pregnancies**

	Cumulative exposure (g) before LMP month and year (N = 1,188). ^a^	Average monthly exposure (g) during LMP month and year (N = 1,091). ^a^
Minimum	2.8 × 10^-4^	1.2 × 10^-4^
5th percentile	3.4 × 10^-1^	1.7 × 10^-2^
10th percentile	1.0	4.0 × 10^-2^
Median	28.9	0.9
75th percentile	117.2	2.9
90th percentile	333.4	7.7
Maximum	3,600	89.2

*Abbreviations:* *LMP* last menstrual period, *PCE* tetrachloroethylene.

^a^There were 1,091 pregnancies that were exposed both before and during the LMP year. All pregnancies exposed during the LMP year were also exposed before the LMP year, but 97 pregnancies exposed before the LMP year were unexposed during the LMP year. Thus, average monthly exposure during the LMP year includes 1,188 exposed pregnancies (1,091 + 97).

We did not observe an association between average monthly PCE exposure during the LMP year and report of at least one ischemic placental disease, holding covariates constant (for PCE ≥ the sample median vs. 0, RR: 0.90; 95% CI: 0.65, 1.24) (Table 4). Alternative classification of average monthly PCE exposure during the LMP year using quartiles or with respect to the 1980 action level of 40 ppb yielded similar findings.

**Table 4:** **Risk ratios and 95% confidence intervals for the association between average monthly PCE exposure (g) during the LMP year and overall ischemic placental disease** ^**a**^ **among 2,110 index pregnancies**

		Model 1: Unadjusted GEE		Model 2: Multivariable GEE ^b^	
	Events/N	RR	95% CI	RR	95% CI
Exposure categorized by any or the sample median					
Any exposure	92/1,091	0.81	0.61, 1.07	0.86	0.65, 1.14
≥50th (5.7 × 10^-1^ g - 89.2 g)	55/635	0.82	0.59, 1.14	0.90	0.65, 1.24
>0- < 50th (1.2 × 10^-4^ g – 5.7 × 10^-1^ g)	37/456	0.79	0.54, 1.15	0.82	0.55, 1.23
0	104/1,019	1.00	Referent	1.00	Referent
Exposure categorized by 1980 action level (40 ppb)					
≥1.136 g (40 ppb)	39/480	0.77	0.54, 1.11	0.88	0.62, 1.26
>0 - <1.136 g	53/611	0.83	0.60, 1.16	0.85	0.61, 1.20
0	104/1,019	1.00	Referent	1.00	Referent
Exposure categorized in quartiles (percentiles)					
≥75th (2.3 g - 89.2 g)	26/331	0.77	0.50, 1.17	0.87	0.56, 1.35
50th-75th (5.7 × 10^-1^ g - 2.3 g)	29/304	0.88	0.57, 1.35	0.91	0.60, 1.39
25th-50th (1.3 × 10^-1^ g - 5.7 × 10^-1^ g)	20/261	0.72	0.44, 1.18	0.74	0.43, 1.26
>0- < 25th (1.2 × 10^-4^ g – 1.3 × 10^-1^ g)	17/195	0.89	0.54, 1.46	0.95	0.58, 1.54
0	104/1,019	1.00	Referent	1.00	Referent

*Abbreviations:* *CI* confidence interval, *GEE* generalized estimating equation, *LMP* last menstrual period, *PCE* tetrachloroethylene, *RR* risk ratio.

^a^Including placental abruption, preeclampsia, small for gestational age, and stillbirth at ≥27 weeks gestation.

^b^Adjusted for maternal age during LMP year (<25 y, 25–29.9 y, 30–34.9 y, 35+ y), year pregnancy ended (<1974, 1975–1980, and >1980), prior ischemic placental disease (nulliparous, parous without history of ischemic placental disease, parous with history of ischemic placental disease), and first-trimester smoking (none, <11 cigarettes/day, ≥11 cigarettes/day).

Next, we examined associations of average monthly PCE exposure during the LMP year and individual ischemic placental diseases. In a sample including both index and non-index pregnancies, we observed 46 cases of placental abruption (1%), 49 cases of preeclampsia (1%), and 27 stillbirths (1%). Vaginal bleeding was present in 7% of pregnancies (N = 326). One-half of pregnancies (N = 23) complicated by placental abruption ended in a preterm birth, as did 12% (N = 6) of preeclampsia cases and 2% (N = 3) of pregnancies that resulted in an SGA infant.

Pregnancies with PCE exposure greater than or equal to the sample median had 2.38-times the risk of ending in stillbirth relative to those with no exposure, holding covariates constant (95% CI: 1.01, 5.59) (Table 5). Pregnancies with the greatest exposure also had a 35% increased risk of placental abruption (95% CI: -32%, 167%) relative to unexposed pregnancies. After restricting to index live births and losses (i.e., the same sample used for analyses of overall ischemic placental disease), pregnancies with prenatal PCE exposure were no more likely to end in placental abruption or stillbirth than unexposed pregnancies (data not shown). Prenatal PCE exposure was not associated with preeclampsia or delivery of an SGA infant. We observed an elevated risk of vaginal bleeding in pregnancies with PCE exposure greater than or equal to the sample median (RR: 1.58; 95% CI: 1.23, 2.02).

**Table 5:** **Risk ratios and 95% confidence intervals for associations between average monthly PCE exposure (g) during the LMP year (percentiles) and individual ischemic placental diseases**

		Model 1: Unadjusted GEE		Model 2: Multivariable GEE ^a^	
	Events/N	RR	95% CI	RR	95% CI
Placental abruption^b^					
Any	18/1,698	1.13	0.63, 2.04	1.19	0.65, 2.15
≥50th (5.7 × 10^-1^ g – 132 g)	11/846	1.36	0.69, 2.70	1.35	0.68, 2.67
>0- < 50th (9.6 × 10^-5^ g – 5.7 × 10^-1^ g)	7/852	0.90	0.41, 1.97	0.90	0.41, 1.97
0	28/3,062	1.00	Referent	1.00	Referent
Preeclampsia^b^					
Any	8/1,721	0.35	0.16, 0.76	0.37	0.17, 0.83
≥50th (5.7 × 10^-1^ g – 132 g)	4/859	0.34	0.12, 0.97	0.36	0.12, 1.07
>0- < 50th (9.6 × 10^-5^ g – 5.7 × 10^-1^ g)	4/862	0.37	0.14, 0.99	0.39	0.14, 1.10
0	41/3,115	1.00	Referent	1.00	Referent
SGA^c^					
Any	68/1,034	0.97	0.69, 1.35	1.04	0.75, 1.46
≥50th (5.7 × 10^-1^ g – 89.2 g)	38/600	0.91	0.61, 1.36	0.98	0.66, 1.45
>0- < 50th (1.2 × 10^-4^ g – 5.7 × 10^-1^ g)	30/434	1.05	0.68, 1.61	1.15	0.75, 1.76
0	62/968	1.00	Referent	1.00	Referent
Stillbirth^b, d^					
Any	13/1,721	1.69	0.77, 3.68	1.67	0.76, 3.67
≥50th (5.7 × 10^-1^ g – 132 g)	9/859	2.39	1.02, 5.61	2.38	1.01, 5.59
>0- < 50th (9.6 × 10^-5^ g – 5.7 × 10^-1^ g)	4/862	0.99	0.31, 3.17	0.98	0.30, 3.14
0	14/3,115	1.00	Referent	1.00	Referent

*Abbreviations:* *CI* confidence interval, *GEE* generalized estimating equation, *LMP* last menstrual period, *PCE* tetrachloroethylene, *RR* risk ratio, *SGA* small for gestational age.

^a^Model for placental abruption adjusted for hypertension before or during pregnancy (yes, no) and first-trimester smoking (none, <11 cigarettes/day, ≥11 cigarettes/day). Model for preeclampsia adjusted for smoking and combined parity and previous ischemic placental disease (nulliparous, parous with no previous ischemic placental disease, parous with previous ischemic placental disease). Model for SGA adjusted for maternal age during LMP year (<25 y, 25–29.9 y, 30–34.9 y, 35+ y), year pregnancy ended (<1974, 1975–1980, and >1980), gestational weight gain <20 lb, hypertension before or during pregnancy, first-trimester smoking, and combined parity and previous ischemic placental disease. Model for stillbirth adjusted for first-trimester smoking.

^b^All live births and stillbirths ≥27 weeks gestation with non-missing data on covariates.

^c^Index live births with non-missing data on covariates.

^d^Stillbirth ≥27 weeks gestation.

Similar effect estimates for overall and individual ischemic placental diseases were obtained when PCE was alternatively modeled as cumulative exposure occurring before the LMP month and year (data not shown). Results were not modified by maternal age, prior ischemic placental disease, year of pregnancy, cigarette smoking, or alcohol use during the first trimester.

## Discussion

Our results suggest that prenatal PCE exposure is not associated with overall ischemic placental disease, but may increase the risk of individual obstetric complications, including stillbirth and placental abruption. This discrepancy between a null overall association despite several positive associations for individual disorders appears due, in part, to the samples used for different analyses. Specifically, prenatal PCE was associated with placental abruption and stillbirth in the full sample of index and non-index pregnancies, but not the smaller sample of pregnancies used for analyses of the composite outcome. We attribute this inconsistency to the small number of exposed cases in the restricted sample rather a systematic difference between the two samples, which came from the same group of mothers.

Of four previous community-based studies relating exposure to PCE-contaminated drinking water to pregnancy loss, only one has reported positive findings. No association was reported in a cross-sectional study of town-level PCE or trichloroethylene (TCE) exposure and fetal loss occurring at >20 weeks gestation in New Jersey women [1], or in a previous publication from this cohort examining associations of prenatal PCE exposure with overall pregnancy loss, first-trimester loss, and combined second- and third-trimester loss [15]. In a study of Woburn, MA residents, women exposed to well water contaminated with PCE, TCE, and other chlorinated organic chemicals were not found to be at an increased risk of pregnancy loss [3]. However, when this analysis was repeated using a more sensitive model of the water distribution system and with data on fetal deaths obtained from the Massachusetts Registry of Vital Records (losses ≥20 weeks or fetuses weighing ≥350 g) rather than self-reported by the participants, women with the greatest exposure had 2.6-times the risk of stillbirth (95% CI: 0.7, 8.9) as unexposed women [2].

Prenatal PCE exposure has been more consistently associated with measures of intrauterine growth restriction. In a retrospective cohort study of Camp Lejeune residents, infants born to PCE-exposed women had a modest elevation in risk of SGA (odds ratio: 1.2, 90% CI: 1.0, 1.3) relative to those of unexposed women, with stronger associations observed in certain vulnerable populations, including women ≥35 years and women with two or more prior losses [4]. Similarly, Endicott, New York women exposed to TCE, but not PCE, in indoor air had 1.2-times the risk (95% CI: 1.0, 1.5) of delivering an SGA infant compared to women from the rest of the state [36]. PCE has been associated with low birth weight (<2,500 g) and very low birth weight (<1,500 g) in some [1, 37], but not all [1, 3, 4, 14, 37], studies. To our knowledge, studies of prenatal PCE with placental abruption and preeclampsia have not been previously undertaken.

PCE exposure assessment was conducted without knowledge of pregnancy complications, making differential exposure misclassification unlikely. Sources of non-differential exposure misclassification include use of 1980 water distribution system conditions to characterize the entire study period and our inability to incorporate individual-level data on water consumption and bathing habits, which were poorly recalled. Despite this, validation studies indicate good correlation of historical PCE sampling with exposure estimates from the original Webler-Brown flow algorithm [38] and the EPANET water distribution system modeling software [39]. Exposure at different time points in gestation may contribute to the exact phenotype observed; however, we were unable to characterize trimester-specific exposure.

SGA was calculated using data from birth certificates, and is therefore unlikely be to misclassified. Women were asked to recall their pregnancies over a period of 19 to 34 years, and may have over- or under-reported placental abruption, preeclampsia, and stillbirth. Previous validation studies suggest moderate to good maternal recall of pregnancy complications [40, 41]. One validation study of women who gave birth 10–15 years earlier reported 94.2% and 96.5% agreement between self-reported preeclampsia and placental abruption with medical records, respectively [40]. Accuracy of recall over longer periods appears similarly acceptable, with Rochester, Minnesota women reporting a previous diagnosis of preeclampsia or eclampsia with 80% sensitivity and 96% specificity over 20 years later [41]. The reliability of women in our cohort is further supported by our finding of good to excellent agreement between questionnaire and birth certificate data for select variables, including birth weight and parity [15]. Any misclassification of pregnancy complications is unlikely to be differential with respect to exposure status, as participants were generally unaware of their exposure to PCE-contaminated drinking water [15].

VL/AC pipes were used to both extend and replace existing pipes, making their distribution within the water system fairly irregular. Consequently, the distribution of PCE exposure was observed to be independent from potentially confounding variables such as age, education, and socio-economic status. However, we cannot rule out the possibility that our results may, in part, be explained by residual or unmeasured confounding. For analyses of stillbirth and preeclampsia, small sample sizes limited the number of covariates we were able to include in adjusted models [42].

Selection bias is unlikely because we were unaware of a woman’s pregnancy complications when deciding her eligibility status, and women who chose not to participate were demographically similar to women who enrolled in the study. We investigated pregnancy complications occurring at 27 weeks gestation or later, and it is possible that a differential loss of pregnancies before this point may have led us to underestimate the true effect size. Specifically, such a ‘competing mortality’ bias could exist if the pregnancies most susceptible to PCE-induced complications of late pregnancy were not included in our analysis because they were also more likely to be spontaneously aborted in the 1st or 2nd trimester [43]. Previous findings from this cohort, however, suggest that prenatal PCE exposure is not associated with early pregnancy loss [15].

Ischemic placental diseases occurring <37 weeks gestation may have a different etiology than those occurring at term. For instance, early onset preeclampsia is thought to be more strongly associated with partial or absent spiral artery remodeling relative to disease occurring at term [44, 45], and prior preterm preeclampsia is more strongly associated with future risk of an ischemic placental disease than previous term preeclampsia [27, 46]. Similar findings have been reported for placental abruption [47]. Although we might therefore expect a stronger association of prenatal PCE exposure with preterm, rather than overall, ischemic placental disease, the small number of preterm births in our cohort precluded us from pursuing this hypothesis. We reported elevated risk ratios for vaginal bleeding at any time during pregnancy. Due to the etiologic heterogeneity of this outcome, we are unable to determine whether bleeding was a marker of placental dysfunction.

## Conclusions

We did not detect an association between prenatal PCE exposure and overall ischemic placental disease. When pregnancy complications were assessed individually, we observed modestly elevated risk ratios for vaginal bleeding, and, in certain samples, stillbirth and placental abruption. Future investigation of drinking-water contamination and ischemic placental disease is necessary, as drinking water contamination remains common [48, 49].

## Electronic supplementary material

Additional file 1: **Eligibility flowchart.** Flowchart showing eligibility criteria and Ns specific to each analysis. (JPEG 62 KB)

## Abbreviations

AC: Asbestos cement
CI: Confidence interval
DEP: Department of Environmental Protection
GEE: Generalized estimating equation
GIS: Geographic information system
LMP: Last menstrual period
PCE: Tetrachloroethylene
RR: Risk ratio
SGA: Small for gestational age
TCE: Trichloroethylene
VL: Vinyl-lined.
