# Supplementary figures and images for: Prenatal drinking-water exposure to tetrachloroethylene and ischemic placental disease: a retrospective cohort study
Source: Environ Health. 2014 Sep 30;13:72. doi: 10.1186/1476-069X-13-72 (PMC4183765; doi:10.1186/1476-069X-13-72)

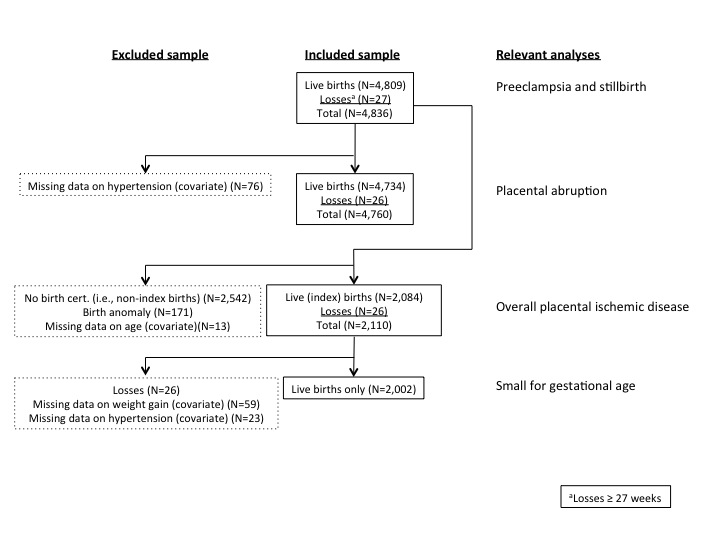

Supplement: Supplementary file 1 — Additional file 1: Eligibility flowchart. Flowchart showing eligibility criteria and Ns specific to each analysis. (JPEG 62 KB) [file 12940_2014_782_MOESM1_ESM.jpeg]
